# Orff Schulwerk in Chinese primary music classrooms: a qualitative study of teachers’ teaching self-efficacy and work engagement

**DOI:** 10.3389/fpsyg.2026.1838857

**Published:** 2026-08-25

**Authors:** SongKun Ge, MengHua Li

**Affiliations:** 1 Faculty of Music, NMA, National Academy of Music “Prof. Pancho Vladigerov”, Sofia, Bulgaria; 2 Department of Musicology, School of Music and Dance, Heze University, Heze, Shandong, Heze, China

**Keywords:** Orff Schulwerk, primary school music teachers, qualitative research, teaching self-efficacy, work engagement

## Abstract

**Introduction:**

While primary school music teachers in China increasingly integrate Orff Schulwerk into their classroom practice, little qualitative research has examined how teachers themselves describe its relationship to their teaching self-efficacy and work engagement.

**Methods:**

Drawing on self-efficacy theory and the Job Demands–Resources model as sensitizing frameworks, this study conducted semi-structured interviews with 18 primary school music teachers in Shandong Province, China, who regularly enacted Orff Schulwerk or Orff-inspired activities. Interview data were analyzed through inductive thematic analysis.

**Results:**

Three main themes were generated. First, teachers’ understandings of Orff Schulwerk converged on diverse pathways of exposure, an experiential interpretation of pedagogical principles, and a phased design of lesson procedures. Second, teachers described their teaching self-efficacy in connection with Orff-informed practice, as reflected in enhanced classroom manageability, increased instructional confidence, more visible learning assessment, and sustained reflection and adjustment. Third, teachers described their work engagement in connection with Orff-informed practice, manifesting in more positive classroom affect, heightened teaching enthusiasm, and a stronger sense of instructional accomplishment.

**Discussion:**

Teachers’ accounts suggest that Orff Schulwerk and its supportive ecology may be experienced as salient occupational resources, and that perceived growth in personal resources such as self-efficacy may accompany the use of this pedagogy. These findings provide contextualized qualitative evidence for the practical significance of Orff Schulwerk in Chinese primary music education and identify plausible pathways for further investigation through mixed-methods or longitudinal research.

## 1 Introduction

Orff Schulwerk is a child-centred pedagogical approach that aims to foster learners’ active participation and creativity through multimodal musical exploration—singing, speaking, playing instruments, moving, and related activities—while emphasizing improvisation and collaborative experiences (Schumacher, 2013; Wang and Sogin, 1997). Compared with traditional music teaching models that prioritize knowledge transmission, Orff Schulwerk aligns with the broader aims of music education to stimulate students’ interest and support holistic development (Bilen, 2010; Martins et al., 2018; Zhang, 2021). Existing studies have documented its benefits for children’s musical development, learning interest, and social interaction skills (Meng, 2023; Salıcı and Eren, 2023; Yun and Kim, 2013).

Within Chinese music education research, Orff Schulwerk has undergone localized adaptation by integrating classroom routines and Chinese musical-cultural resources, and it has been widely implemented at the primary level (Lisha and Banchongsilpa, 2025; K. Wang and Webb, 2024). The approach not only enhances students’ interest but also foregrounds their active engagement and embodied practice, enabling children to develop musical skills and cultivate musical enjoyment and aesthetic sensitivity through diversified activities such as instrumental performance and improvisation (L. Wang and Odena, 2025; Jiang, 2025; Kwon and Lee, 2012). From a teacher perspective, classroom organization characterized by experience-based learning, collaboration, and immediate feedback may improve student engagement, classroom order, and interaction quality, which in turn can strengthen teachers’ perceptions of classroom structure and controllability and shape their work-related affect and work engagement (Dilekçi et al., 2025; Frenzel et al., 2018). Here, work engagement refers to a positive, fulfilling work-related state characterized by vigor, dedication, and absorption (Schaufeli and Bakker, 2004). Such practices may also contribute to higher teaching self-efficacy and a more positive teaching experience (Sabljar et al., 2020; Tschannen-Moran and Hoy, 2001). In addition, Orff Schulwerk can provide a more open classroom climate and greater space for teachers’ self-expression (Ozevin, 2021). Research on teachers’ work has shown that job resources—such as instructional autonomy and supportive organizational environments—are associated with lower work stress and higher job satisfaction (Ouellette et al., 2018; Pearson and Moomaw, 2005). Evidence from teacher education further suggests that participation in Orff-related courses or training may strengthen efficacy beliefs closely tied to classroom enactment (e.g., movement self-efficacy), which co-develop with teachers’ instructional confidence and ongoing adjustments in practice (Fisher and Rose, 2011; Kikoler, 2022). Taken together, existing research suggests that Orff Schulwerk may be relevant not only to students’ learning experiences but also to primary school music teachers’ teaching self-efficacy and work engagement.

Teacher self-efficacy is a robust predictor of teachers’ instructional behaviours and affective orientations. Teachers with stronger self-efficacy often perform better in classroom management, instructional innovation, and emotion regulation (Biasutti et al., 2021; S. Li, 2023). However, under the pressures of curriculum reform, heavy workload, and insufficient resources, primary school teachers commonly face substantial job stress, and some report declines in work engagement and job satisfaction (Sabljar et al., 2020). Among music teachers in particular, non-instructional administrative demands, evaluation- and event-related tasks, and classroom discipline are repeatedly identified as salient sources of stress, which may further undermine occupational well-being and classroom performance (Wong et al., 2019). At the same time, a positive work mindset has been shown to support higher-quality classroom interaction and teacher professional development (Schaufeli and Bakker, 2004). Studies also indicate that among teachers who receive Orff training or other comparable innovative pedagogy training, the frequency of creative, movement-based, and exploratory activities increases significantly (Sogin and Wang, 2008). Such shifts may enhance teachers’ self-efficacy, strengthen their confidence in achieving instructional goals, and encourage the adoption of more diversified teaching strategies (Valdebenito and Almonacid-Fierro, 2022; Zainal and Matore, 2021).

To situate teachers’ work engagement within a broader work-context framework, this study draws on the Job Demands–Resources (JD–R) model. The JD–R model conceptualizes the work environment in terms of job demands and job resources (Bakker and Demerouti, 2007). Excessive job demands—such as time pressure, emotional labour, and workload—are more likely to trigger depletion and burnout, whereas job resources—such as autonomy, supportive relationships, teaching resources, and professional development opportunities—promote work engagement through a motivational pathway and can buffer the negative impact of high demands (Bakker and Demerouti, 2017; Demerouti et al., 2001). In teacher research, the JD–R model has been widely used to explain associations among teachers’ work engagement, occupational stress, and well-being/mental health (Hakanen et al., 2006; Y. Li et al., 2025). Importantly, the model also highlights the role of personal resources in motivational processes: self-efficacy, optimism, and organization-based self-esteem are positively associated with work engagement and may amplify the benefits of job resources or mitigate the adverse effects of high demands (Xanthopoulou et al., 2007, 2009). Empirical studies in educational contexts further support links between personal resources (including self-efficacy) and teacher work engagement (Sheridan et al., 2025). Moreover, evidence suggests that when job demands are high (e.g., intensive classroom behaviour management), job resources become particularly critical for sustaining teacher engagement, consistent with the buffering and motivational mechanisms proposed by the JD–R model (Bakker et al., 2007). From this perspective, Orff Schulwerk may be conceptualized as a bundle of job resources by increasing instructional autonomy, enriching task variety, and enhancing social and performance feedback through collaborative music-making. Related training and school-based teaching research activities can further function as developmental resources, while strengthened teaching self-efficacy represents a key personal resource through which these job resources may translate into higher work engagement.

Against this backdrop, research on Orff Schulwerk remains limited. Much of the existing literature focuses on music teachers or students in Western contexts, with relatively little in-depth analysis of Chinese teachers (L. Wang and Odena, 2025; Xiong, 2021). In addition, prior studies often examine teaching self-efficacy or work engagement separately, and few have systematically integrated both constructs to examine how teachers describe Orff-informed practice in relation to their occupational psychological experiences (Hernández-Micó and Domínguez-Lloria, 2024).

Self-efficacy theory posits that individuals’ behaviours, motivation, and emotional reactions are shaped largely by beliefs about their capabilities, emphasizing confidence and self-evaluation regarding the successful completion of tasks (Bandura, 1997; Bandura and Adams, 1977). This perspective has been widely adopted to explain teachers’ instructional beliefs, motivational orientations, and affective attitudes (Klassen and Chiu, 2010; Zee and Koomen, 2016). Prior research also indicates a positive association between self-efficacy and work engagement: stronger self-efficacy can enhance teachers’ confidence in achieving instructional goals and increase enthusiasm and occupational investment (Schiavio et al., 2020; Woods and Copur-Gencturk, 2024). In music education research, music teacher self-efficacy has been recognized as an independent psychological construct that positively predicts teaching creativity, classroom interaction, and student learning outcomes (Concina, 2023; Nie et al., 2013). Existing studies of Orff Schulwerk often take classroom activity types and instructional enactment as primary analytic units, describing and comparing the musical activities adopted and their organization (Eren and Gül, 2017). Particularly in Chinese primary music education, systematic investigations of how teachers describe their teaching self-efficacy and work engagement in relation to Orff-informed practice remain scarce, limiting a comprehensive understanding of its broader implications for teachers’ occupational psychological experiences (Guo, 2018; Shiyao and Noordin, 2024; Yang and Welch, 2023).

Accordingly, grounded in self-efficacy theory and the JD–R model, this qualitative study employs semi-structured interviews and thematic analysis to examine Chinese primary school music teachers who implement Orff Schulwerk. The study aims to (a) explore how Chinese primary school music teachers describe their teaching self-efficacy and work engagement in relation to Orff-informed practice, and (b) identify perceived associations, interpretive pathways, and contextual factors reflected in teachers’ narratives.

This study contributes to the literature in three ways. First, prior research on Orff Schulwerk has predominantly examined either student-level outcomes (musical skills, social–emotional development) or, where teachers are the focus, has treated teaching self-efficacy and work engagement as separate constructs (Concina, 2023; Hernández-Micó and Domínguez-Lloria, 2024; L. Wang and Odena, 2025). To our knowledge, no prior qualitative study has integrated these two constructs to examine how teachers themselves narrate the connection between a specific pedagogy and their occupational psychological experiences. Second, although Orff Schulwerk has been widely adopted in Chinese primary music classrooms, in-depth qualitative accounts from Chinese teachers remain scarce, with existing Chinese-context studies relying largely on descriptive or document-based analyses (Guo, 2018; Yang and Welch, 2023). Third, by drawing on self-efficacy theory and the Job Demands–Resources model as sensitizing frameworks while allowing themes to be generated inductively from teachers’ accounts, the study offers a context-specific, process-oriented account of how a pedagogy may be appropriated as a lived occupational resource, and contributes interpretive evidence that complements existing variable-centred research.

The study addresses the following research questions:

*RQ1:* How do Chinese primary school music teachers understand and enact Orff Schulwerk in their classroom practice?

*RQ2:* How do Chinese primary school music teachers describe their teaching self-efficacy in relation to the use of Orff Schulwerk?

*RQ3:* How do Chinese primary school music teachers describe their work engagement in relation to the use of Orff Schulwerk?

## 2 Materials and methods

This study adopted a qualitative design to explore how Orff Schulwerk relates to Chinese primary school music teachers’ teaching self-efficacy and work engagement. Given the focus on teachers’ understandings of Orff Schulwerk, their classroom enactment experiences, and the meaning-making processes surrounding subjective psychological experiences (e.g., efficacy beliefs and work-related states), qualitative interviewing was well suited to capturing experiential accounts of “how” and “why.” Interview data were analyzed using thematic analysis following Braun and Clarke’s (2006) six-phase approach to ensure a systematic and transparent coding and theme-development process.

### 2.1 Participants and recruitment

Purposive sampling combined with snowball recruitment was used to identify primary school music teachers in Shandong Province, China, with substantive Orff-related teaching experience.

Inclusion criteria were: (a) current employment as a primary school music teacher in Shandong Province; (b) regular use of Orff Schulwerk or Orff-inspired activities in classroom instruction, defined as recurring incorporation of Orff or Orff-inspired activities into teaching rather than mere awareness of the approach or a one-off trial; and (c) prior formal exposure to Orff Schulwerk through at least one of the following routes: pre-service teacher education focused on or substantively including Orff Schulwerk, in-service training programmes led by certified or experienced Orff practitioners, university coursework focused on Orff Schulwerk, or sustained participation in school-based jiaoyan (teaching-research) activities centred on Orff Schulwerk. Recruitment proceeded through professional networks and snowball sampling, with the first author contacting potential participants and confirming eligibility through a pre-interview screening conversation that asked about training history, current frequency of Orff-informed practice, and willingness to reflect in depth on classroom experience.

Eighteen primary school music teachers participated. The sample included nine female and nine male teachers, ranging in age from 29 to 52 years (M = 34.4 years). Thirteen teachers held a bachelor’s degree and five held a master’s degree. Teaching experience ranged from 3 to 32 years (M = 10.4 years). Sixteen teachers were employed in public primary schools and two in private primary schools, providing variation in organizational and resource contexts (see Table 1).

**Table 1:** Participants’ information.

Participant	Gender	Age	Educational background	Years of teaching experience	School type
T1	Male	30	Master’s	7 years	Private
T2	Male	30	Bachelor’s	3 years	Public
T3	Male	35	Bachelor’s	12 years	Public
T4	Female	35	Bachelor’s	7 years	Private
T5	Male	31	Bachelor’s	7 years	Public
T6	Male	29	Bachelor’s	5 years	Public
T7	Female	37	Bachelor’s	14 years	Public
T8	Female	35	Master’s	9 years	Public
T9	Female	39	Bachelor’s	15 years	Public
T10	Male	40	Bachelor’s	18 years	Public
T11	Male	29	Bachelor’s	7 years	Public
T12	Female	37	Bachelor’s	12 years	Public
T13	Male	33	Bachelor’s	9 years	Public
T14	Female	35	Master’s	12 years	Public
T15	Female	31	Bachelor’s	9 years	Public
T16	Female	52	Bachelor’s	32 years	Public
T17	Male	29	Master’s	3 years	Public
T18	Female	33	Master’s	6 years	Public

### 2.2 Ethical statement

The study was granted an exemption from full ethics review by the Academic Committee of the School of Music and Dance, Heze University, on the grounds that it involved adult participants who provided voluntary written informed consent, used anonymized data, and posed no foreseeable risks beyond those of ordinary academic conversation.

All participants provided written informed consent prior to data collection. They were informed of the study purpose, the confidentiality and anonymization procedures, the voluntary nature of participation, and their right to withdraw at any time without penalty. With participants’ permission, interviews were audio-recorded. Data were anonymized during transcription, with pseudonyms (T1–T18) replacing personal identifiers and school names. All materials were stored on a password-protected institutional drive accessible only to the research team and were used solely for academic research purposes.

### 2.3 Data collection

Data were collected between 4 July and 25 August 2025 through semi-structured in-depth interviews conducted via online audio calls. Interviews lasted approximately 45–60 min (M = 52 min) and were conducted in Mandarin Chinese by the first author. All interviews were audio-recorded with participants’ written consent and subsequently transcribed verbatim by the first author, with transcripts checked against the recordings for accuracy.

#### 2.3.1 Interview protocol development

The interview guide was developed iteratively. An initial draft was generated from the two theoretical frameworks (self-efficacy theory and the JD–R model) and from prior qualitative studies of music teachers and Orff Schulwerk practitioners (Locke, 2016; Ozevin, 2021; Pellegrino, 2015). The draft was refined through consultation with the corresponding author and one additional senior researcher with expertise in qualitative methodology, with revisions focused on the openness of question phrasing and the sequencing of topic domains. The revised guide was then pilot-tested with primary school music teachers who met the inclusion criteria but were not included in the final sample; based on the pilot, several questions were merged and additional probing prompts were added to elicit accounts of difficulty and ambivalence alongside positive experiences.

#### 2.3.2 Topic domains

The final interview guide covered five topic domains: (1) teachers’ understanding and classroom enactment of Orff Schulwerk; (2) teachers’ teaching self-efficacy in connection with Orff-informed practice; (3) teachers’ work engagement in connection with Orff-informed practice; (4) the perceived relationship between teaching self-efficacy and work engagement in the context of Orff-informed practice; and (5) implementation challenges and recommendations for early-career colleagues. Within each domain, the interviewer used open-ended primary questions and follow-up probes (for example, “Could you describe a specific lesson where this happened?” “What did that feel like for you?” “Have you ever had the opposite experience?”) rather than reading questions verbatim, and question order was adjusted in response to participants’ narratives. The complete interview guide is provided as Supplementary Appendix A.

#### 2.3.3 Trustworthiness procedures

Several procedures supported trustworthiness during data collection and early analysis: the first author maintained reflective notes after each interview to track emerging interpretive ideas and to flag affective tone and probable interpretive bias; transcripts were checked against the recordings; and the two researchers conducted ongoing discussion of interview content and emerging coding ideas during the analytic process. Thick description with representative quotes is used in the Results section to enable readers to evaluate the evidentiary basis for each theme.

### 2.4 Data analysis

Transcripts were imported into NVivo 15 for coding and analytic management. Data were analyzed through thematic analysis following Braun and Clarke (2006, 2019, 2021) six-phase procedure.

#### 2.4.1 Analytic stance

The analysis combined a deductive organizing layer with inductive theme generation, following the hybrid approach of Fereday and Muir-Cochrane (2006). Deductively, the domain-level architecture of the analysis was fixed in advance: because the three research questions correspond to three sensitizing domains (understandings and enactment of Orff Schulwerk; teaching self-efficacy; work engagement), the reporting of themes is organized under three main themes that mirror these domains, and the interview protocol was structured around the same domains. Inductively, everything within that architecture was generated from the data: the codes were produced through line-by-line open coding without an *a priori* coding template, and the 10 subthemes were formed bottom-up by aggregating semantically related codes, rather than being derived from the constructs’ theoretical dimensions. Self-efficacy theory and the JD–R model thus informed what we asked about and how the findings are framed in the Discussion, but not how segments of data were coded or how subthemes were formed.

#### 2.4.2 Coding level

Coding was predominantly semantic, capturing the surface meanings expressed by participants. Limited latent coding was used where teachers’ accounts referenced affective evaluations or implicit assumptions about the classroom (for example, an unspoken contrast between Orff-informed and traditional instructional approaches).

#### 2.4.3 Code generation

Two researchers (the first author and one additional researcher) independently conducted line-by-line open coding of all 18 transcripts. In-vivo codes were preferred where teachers’ wording was vivid and concept-bearing (for example, “a shot of confidence,” “the children’s eyes light up”). The initial round of open coding generated approximately 90 codes across the two coders’ code lists.

#### 2.4.4 Code-to-theme procedure

Codes from the two coders were compared and consolidated through structured discussion. Semantically close codes were aggregated into candidate subthemes; the resulting subthemes were then allocated to the three domain-level main themes described above. The content and boundaries of each subtheme were determined by the coded data rather than by theoretical expectation: subthemes that did not map onto any construct dimension (for example, the phased design of lesson procedures, or the visibility of student learning during performance-based activities) were retained on the strength of their grounding in the data. Each candidate theme was iteratively tested against the full dataset to ensure that it was both internally coherent and distinct from other themes. Definitions, representative codes, and illustrative quotes for each subtheme are summarized in Table 2.

**Table 2:** Themes, subthemes, definitions, representative codes, and illustrative quotes from the thematic analysis.

Main theme	Subtheme	Definition	Representative codes (open coding)	Illustrative quote
Theme 1. Teachers’ understanding and enactment of Orff Schulwerk *(RQ1)*				
	Diverse pathways of exposure	Teachers encountered Orff Schulwerk through multiple, overlapping channels rather than through a single formal route.	Face-to-face training; in-service workshops; online courses; jiaoyan (teaching-research) activities; collegial sharing; peer learning	“Sometimes it’s onsite training… I’ve also taken some online courses… now we have unified training for professional development, and I selected some Orff-related courses.” (T8)
	Experiential understanding of pedagogical principles	Teachers interpreted Orff Schulwerk as an experiential, multimodal, child-centred orientation rather than a fixed lesson template.	Elemental music; integration of speech, body, movement, singing, instruments; child experience as starting point; learning while playing	“It’s the approach that best restores music’s elemental nature… it integrates language, bodily movement, singing, dance, and instrumental playing.” (T6)
	Phased design of lesson procedures	Teachers translated Orff principles into a recognizable phased sequence of classroom activities.	Listen → imitate → group work → creation; stepwise organization; flexible integration with Kodály and Curwen hand signs	“Let the children listen first, then imitate… and the next step is to work in groups… continuously creating alarm-clock shapes.” (T3)
Theme 2. Teaching self-efficacy described in relation to Orff Schulwerk *(RQ2)*				
	Enhanced classroom manageability	Teachers reported perceiving greater control over classroom behaviour and routines through music-game-based regulation.	Transfer of game rules to behaviour; reduced management demand; teacher–student tacit coordination; smoother routines over time	“In the games… it increases control over physical functioning and emotions… so over time my class became easier to manage.” (T3)
	Increased instructional confidence	Teachers described growing confidence in their teaching capability through accumulated practice and trial-and-error refinement.	Repeated practice; stable expectations; faster response; coming through the “mess”; gradually finding methods	“I feel more confident in my teaching because of continuous practice… when I give a signal, the children respond quickly… then the activities can run faster and better.” (T3)
	More visible assessment	Performance- and movement-based activities rendered students’ learning more directly observable, supporting timely adjustment.	Visible engagement; spot weak points; immediate documentation; multidimensional feedback; targeted adjustment next semester	“Through music games or movement… you can see where they are weak… the teacher keeps observing and records immediately… it helps make targeted adjustments next semester.” (T3)
	Sustained reflection and adjustment	Teachers reported ongoing in-the-moment and across-lesson reflection on activity difficulty, role allocation, and student differences.	Grasping the “degree”; calibration to student age; role assignment for reluctant students; scaffolding; combining modalities	“You have to grasp the right ‘degree’… and adjust according to students’ age… not every lesson is successful… you can make changes, such as combining movement with music, pictures, and so on.” (T5)
Theme 3. Work engagement described in relation to Orff Schulwerk *(RQ3)*				
	Enhanced positive affect	Teachers described moments of shared immersion and emotional satisfaction during Orff-informed lessons.	Whole-self immersion; exhilaration; playing together with children; sense of self-worth tied to coordinated student performance	“I feel like my whole self is in that atmosphere… it feels exhilarating… playing together with the children.” (T3)
	Increased teaching enthusiasm	Teachers framed Orff-informed practice as a source of motivation for sustained learning and instructional exploration.	Documenting practice in cases; continuous reading; integration with Kodály; persistent adaptation to Chinese textbooks	“I write my daily teaching into cases… keep reading… and I’m also exploring Kodály pedagogy… the two approaches can be combined and complement each other.” (T3)
	Strengthened instructional accomplishment	Teachers connected a sense of accomplishment to immediate positive student feedback during and after class.	Bright eyes; rushing to greet teacher; post-class closeness; helping with instruments; perception of time flowing quickly	“The children’s eyes are wide open and bright… I feel a great sense of accomplishment… after class they rush to greet you.” (T3)

*N* = 18 primary school music teachers, Shandong Province, China.

Themes were generated inductively from line-by-line open coding of 18 semi-structured interview transcripts in NVivo 15, with self-efficacy theory and the Job Demands–Resources model serving as sensitizing concepts rather than as an a priori coding template. Each subtheme paragraph in Section 3 (Results) includes a coverage statement of the form “(n out of 18 participants).” Illustrative quotes have been minimally tidied for readability without altering meaning.

#### 2.4.5 Intercoder agreement and disagreement handling

The analytic approach was consensus-oriented (codebook/team-based thematic analysis) rather than reliability-oriented. Consistent with this tradition, we did not calculate Cohen’s *κ*; the analytic goal was to develop a shared and defensible coding structure through structured discussion of disagreements rather than to estimate inter-rater agreement statistically (Braun and Clarke, 2019, 2021; McDonald et al., 2019). All discrepancies between the two coders were logged in NVivo memos and resolved through structured consensus discussion, with occasional consultation of the corresponding author for residual interpretive disagreements. The final coding structure was re-applied to the full dataset by both researchers to verify evidentiary support for every subtheme.

Software. NVivo 15 was used to manage transcripts and audio files, organize codes and memos, support cross-case comparison during theme refinement, and maintain a complete audit trail of analytic decisions. NVivo’s automatic sentiment coding was not used in theme generation.

### 2.5 Researcher reflexivity

The first author is a doctoral candidate in music education with prior personal experience of Orff Schulwerk through professional training and primary classroom teaching. This background informed the design of the interview protocol and facilitated rapport with participants, and we acknowledge that it plausibly shaped the interpretation of teachers’ accounts—particularly through a predisposition to attend closely to positive experiences of Orff-informed practice and to recognize Orff-specific vocabulary and analogies in participants’ narratives.

To manage this predisposition, several steps were taken throughout the study. During interviewing, the first author included open prompts that explicitly invited descriptions of difficulties, unsuccessful attempts, and contexts in which Orff-informed practice felt unsuitable, and avoided leading framings of teachers’ experiences. During analysis, reflective notes were maintained alongside coding to surface and examine the researchers’ interpretive tendencies, and codes that appeared to reflect researcher preference rather than participant meaning were flagged and revisited. The second author—whose music education background does not include extensive Orff-specific training—served as an external check on interpretations grounded too strongly in Orff-specific assumptions during the consensus-coding discussions. Methodological decisions, including why particular codes were aggregated, split, or discarded, were documented in NVivo memos.

The transferability boundaries arising from this positionality, together with the purposive sampling design and the self-report nature of the data, are taken up explicitly in the Discussion and Limitations sections.

## 3 Results

The thematic analysis generated three main themes and 10 subthemes corresponding to the three research questions. Table 2 provides an overview of the themes, subthemes, coverage across participants, definitions, representative open codes, and illustrative quotations. The narrative results below elaborate each theme and subtheme, with coverage statements indicating how broadly each subtheme was shared across the 18 participants. Because the study used a qualitative cross-sectional design, the findings are presented as teachers’ perceptions, experiences, and meaning-making rather than as evidence of causal effects.

### 3.1 RQ1. How do Chinese primary school music teachers understand and enact Orff Schulwerk in their classroom practice?

The analysis suggests that teachers’ understandings and enactments of Orff Schulwerk clustered around three interrelated aspects: diverse pathways of exposure, an experiential understanding of pedagogical principles, and a phased design of lesson procedures.

Diverse pathways of exposure. This subtheme was reflected in the accounts of most of the 18 participants. Teachers commonly described multiple routes through which they encountered Orff Schulwerk. One route involved more formal and structured professional learning, including face-to-face training, teaching-research and professional development programmes, and online courses. As T8 noted, their learning experiences combined onsite training with online and programme-based learning:

*“Sometimes it’s onsite training… and I’ve also taken some online courses… now we have unified training for professional development, and I selected some Orff-related courses.”* (T8).

Another route reflected school culture and collegial learning. Some teachers reported a shared atmosphere of ongoing inquiry within their schools and further deepened their understanding through external training opportunities. T1 recalled the emphasis placed on Orff Schulwerk in their private school:

*“At our private school, the music teachers are all studying it… the school values Orff Schulwerk more, and we often go to Shenzhen, Hangzhou, and other places across the country for training and learning.”* (T1).

Experiential understanding of pedagogical principles. This subtheme was reflected in the accounts of nearly all of the 18 participants. At the level of pedagogical principles, teachers often viewed Orff Schulwerk as capturing the “elemental” and holistic nature of music through the integration of speech, bodily movement, singing, dance, and instrumental performance, enabling students to develop musical understanding through participation. T6 emphasized its integrative character:

*“It’s the approach that best restores music’s elemental nature… it integrates language, bodily movement, singing, dance, and instrumental playing.”* (T6).

Other teachers interpreted Orff principles from the perspective of children’s learning experiences, highlighting the child’s immediate perception as the starting point and making learning more concrete and less abstract. T9 explained:

*“It’s mainly based on children’s experiences… through clapping and body movement, it becomes more vivid and less abstract… they can learn it while playing.”* (T9).

Phased design of lesson procedures. This subtheme was reflected in the accounts of a majority of the 18 participants. In describing classroom enactment, teachers’ accounts converged on a phased instructional sequence: lessons typically began with listening and sensing, moved through imitation and reproduction, and gradually progressed toward collaborative creation and expression. Using a “clock/alarm clock” themed activity as an example, T3 described a stepwise organization:

*“Let the children listen first, then imitate… and the next step is to work in groups… continuously creating alarm-clock shapes.”* (T3).

Teachers also reported flexibly integrating multiple strategies according to instructional goals and students’ prior knowledge when addressing musical elements. For example, T6 described combining other approaches during pitch learning:

*“Children freely form groups… take turns introducing their names… and for pitch learning… before teaching a new song, I use Curwen hand signs in the air to show the melodic contour and help them build pitch concepts.”* (T6).

Overall, teachers’ understandings of Orff Schulwerk were described as developing through multi-channel professional learning and supportive school cultures, alongside an endorsement of experiential learning and the construction of actionable lesson procedures.

### 3.2 RQ2. How do Chinese primary school music teachers describe their teaching self-efficacy in relation to the use of Orff Schulwerk?

Teachers described their teaching self-efficacy in close connection with Orff-informed classroom practice, reflected in four subthemes: classroom manageability, instructional confidence, visible assessment, and sustained pedagogical reflection and adjustment.

Classroom manageability. This subtheme was reflected in the accounts of most of the 18 participants. Teachers described classroom manageability in connection with Orff-informed activities, particularly music games through which students practised self-regulation, rule awareness, and coordinated participation. As T3 noted:

*“In the games… it increases control over physical functioning and emotions… so over time my class became easier to manage… it’s closely related to following the game rules.”* (T3).

Teachers also described that, with accumulated practice and increased familiarity with students, organizing and regulating activities became smoother. This was often narrated as a sense of “tacit coordination” between teachers and students. T12 stated:

*“At the beginning it was difficult… but now that we have developed a tacit understanding… with tasks assigned for each child… their attention is strong and the classroom atmosphere is very good.”* (T12).

Instructional confidence. This subtheme was reflected in the accounts of nearly all of the 18 participants. Teachers described instructional confidence as developing alongside sustained practice, trial and error, and iterative refinement, as they formed more stable expectations regarding classroom routines and activity outcomes. T4 reflected on a trajectory from early “chaos” to gradually identifying effective strategies and achieving more stable classroom effects:

*“At first the outcomes* var*ied a lot… it depended on classroom control and students’ level… I also came through the ‘mess’… I gradually found methods, and now it feels pretty good.”* (T4).

This account illustrates how teachers associated confidence not with a simple or immediate effect of Orff-informed practice, but with repeated experimentation, adjustment, and familiarity with students’ responses over time.

Visible assessment. This subtheme was reflected in the accounts of most of the 18 participants. Teachers emphasized that performance-based activities, often embedded in group work, movement, and music games, provided opportunities to directly observe students’ participation, skill development, and areas of difficulty, and to adjust instruction accordingly. T3 noted:

*“Through music games or movement… you can see where they are weak… the teacher keeps observing and records immediately… it helps make targeted adjustments next semester.”* (T3).

Some teachers indicated that they used such practices less frequently or felt their understanding was still developing, yet they still perceived differences from conventional instruction. T16 stated:

*“I can only say it’s relatively direct… not fully comprehensive… I use it less, but I can see the changes compared with traditional methods.”* (T16).

Sustained reflection and adjustment. This subtheme was reflected in the accounts of most of the 18 participants. Most teachers described an ongoing orientation toward reflection and in-the-moment adjustment. On the one hand, they adjusted activity difficulty, formats, and materials based on classroom effects and student feedback; on the other hand, they attended to individual differences by allocating roles and providing scaffolding to increase participation. T5 highlighted the need to calibrate activities and adjust in response to students’ age and classroom dynamics:

*“You have to grasp the right ‘degree’… and adjust according to students’ age… not every lesson is successful… you can make changes, such as combining movement with music, pictures, and so on.”* (T5).

T12 described supporting students who initially struggled to participate by observing them and assigning suitable roles, gradually facilitating fuller engagement:

*“At first some students definitely could not join in… after observing, you assign them a role that fits… and then gradually let them express themselves more.”* (T12).

Taken together, teachers’ accounts indicate that perceived growth in their teaching self-efficacy was described in close connection with Orff-informed practice across four interrelated subthemes: classroom manageability, instructional confidence, visible assessment, and sustained reflection.

### 3.3 RQ3. How do Chinese primary school music teachers describe their work engagement in relation to the use of Orff Schulwerk?

Teachers’ accounts of classroom practice and emotional experience described work engagement in connection with Orff-informed practice. Three subthemes were identified: positive classroom affect, teaching enthusiasm, and instructional accomplishment.

Positive classroom affect. This subtheme was reflected in the accounts of most of the 18 participants. Many teachers described emotional satisfaction when they and students became jointly immersed in musical activities during Orff-informed lessons. Teachers often connected such affective experience with visible student participation and perceived instructional effectiveness. T15 noted that when students could perform movement tasks in a coordinated manner, this was accompanied by stronger confidence and a sense of self-worth:

*“When I finish teaching a sequence of movements and the students do them neatly… that confidence and sense of self-worth comes out… it’s very useful for primary school music classes.”* (T15).

This account shows how teachers described positive affect as emerging through concrete classroom interactions, especially when students’ coordinated participation made the lesson feel meaningful and successful.

Teaching enthusiasm. This subtheme was reflected in the accounts of most of the 18 participants. Teachers’ accounts suggested that Orff-informed practice co-occurred with efforts to move beyond established routines and to improve lesson design through active reflection, reading, and continuous learning, including the integration of multiple pedagogical approaches. Teachers also emphasized that integrating Orff principles into Chinese textbooks was not a matter of direct transplantation but required sustained learning and adaptation. As T14 noted:

*“Not every piece is suitable for Orff… you need continuous learning to apply these methods to the textbooks and integrate them… it’s challenging and requires persistence.”* (T14).

This emphasis on persistence and adaptation suggests that teachers framed enthusiasm not merely as momentary enjoyment, but also as a continuing willingness to learn, experiment, and refine their practice.

Instructional accomplishment. This subtheme was reflected in the accounts of nearly all of the 18 participants. Teachers commonly connected a sense of accomplishment to immediate and positive student feedback, such as high concentration during class, spontaneous interaction after class, and expressions of closeness. T7 suggested that students’ enjoyment was associated with teachers’ own positive teaching experience, making class time feel faster and teaching feel more meaningful:

*“The children are very happy… they can move, sing, and dance… and I feel time passes quickly—class ends before I know it.”* (T7).

Such accounts suggest that teachers’ sense of instructional accomplishment was closely tied to their perception of students’ enjoyment, participation, and responsiveness during Orff-informed activities.

Overall, teachers’ accounts indicate that work engagement was described in close connection with Orff-informed practice through three interrelated subthemes: positive classroom affect, teaching enthusiasm, and instructional accomplishment. These findings should be interpreted as teachers’ perceived experiences and meaning-making rather than as evidence of causal effects.

### 3.4 Challenges and boundary conditions in Orff-informed practice

Although the three main themes above are predominantly positive in tone, the interviews also contained accounts of difficulty and ambivalence, and these ran through several of the subthemes reported above. Consolidating them here provides a fuller picture of how teachers experienced the integration of Orff Schulwerk into their everyday work.

Three recurrent strands of challenge can be identified in the accounts already quoted. First, early-stage implementation was frequently described as disorderly and effortful: teachers recalled initial periods in which classroom outcomes varied considerably and organization was difficult (T4: “came through the ‘mess’”; T12: “at the beginning it was difficult”), with a workable routine emerging only after repeated experimentation. Second, teachers described persistent calibration demands rather than a settled method: activities had to be continuously adjusted to students’ age and ability differences as well as to classroom dynamics (T5: “not every lesson is successful”), and some students initially could not participate at all and required individually assigned roles before engaging (T12). Third, several teachers pointed to limits of the approach itself: the fit between Orff-informed activities and the prescribed Chinese textbook repertoire was described as partial and effortful to achieve (T14: “not every piece is suitable for Orff”), and some teachers reported using performance-based assessment practices selectively or less frequently while their understanding was still developing (T16).

No participant, however, described abandoning Orff-informed practice or judging it unsuitable for their context overall; difficulty was consistently narrated as an early-stage or ongoing calibration problem rather than as grounds for discontinuation. Given that the sample was purposively drawn from teachers with sustained Orff-related engagement, this pattern should be read with the self-selection boundary discussed in the Limitations section in mind: teachers who attempted Orff-informed teaching and discontinued it are not represented here, and their absence likely understates the prevalence and severity of implementation difficulty in the wider teaching population.

## 4 Discussion

Guided by self-efficacy theory and the Job Demands–Resources (JD–R) model, this study drew on semi-structured interviews with 18 primary school music teachers in Shandong Province to examine how Orff Schulwerk is enacted in Chinese primary music classrooms and how it relates to teachers’ teaching self-efficacy and work engagement. Thematic analysis addressed three research questions concerning teachers’ understandings of Orff Schulwerk (RQ1), how teachers described teaching self-efficacy in relation to Orff-informed practice (RQ2), and how teachers described work engagement in relation to Orff-informed practice (RQ3). Overall, the findings provide narrative-based, process-oriented evidence for links among pedagogy, school context, and teachers’ psychological experiences, and offer a complementary lens for understanding how a pedagogy may be translated into teachers’ lived experiences of resources in real classrooms (Carrillo et al., 2015; Kelly-McHale, 2013).

### 4.1 Teachers’ understandings of Orff Schulwerk (RQ1)

The findings indicate that teachers’ understandings of Orff Schulwerk comprised three interrelated elements: diversified pathways of exposure and learning (e.g., training, teaching-research activities, classroom observation, and online courses), endorsement of a “child-experience-centred” pedagogical orientation, and a structured understanding of lesson procedures organized progressively from “listening” to “imitation” and then “creation.” This pattern suggests that teachers did not treat Orff Schulwerk merely as a labeled method; rather, they translated it into actionable classroom logics and design frameworks. This aligns with narrative-based research on music teachers, which suggests that pedagogical approaches are often appropriated as resources that support classroom organization, instructional decision-making, and professional identity construction, rather than functioning solely as technical tools (Beijaard et al., 2004; Kelchtermans, 2009).

At the level of pedagogical principles, teachers emphasized that Orff Schulwerk supports student engagement and creative experience through multimodal, movement-based, and collaborative activity structures (Elkoshi, 2024). Such an experiential, participatory, and integrative understanding also resonates with broader expectations for music classrooms within the discourse of quality-oriented education (Eren et al., 2013; Şeker and Bilen, 2010). Importantly, teachers’ “proceduralized” understanding implies an ability to operationalize pedagogical principles into concrete instructional actions and to obtain observable student responses through interaction. These experiences may provide a practical foundation for subsequent shifts in efficacy beliefs and work engagement.

### 4.2 Teaching self-efficacy in relation to Orff-informed practice (RQ2)

Teachers’ accounts linked Orff-informed practice with four perceived aspects of teaching self-efficacy: greater classroom manageability, increased instructional confidence, more visible assessment, and sustained reflection and adjustment. These patterns are consistent with a core proposition of self-efficacy theory, namely that efficacy beliefs are shaped by individuals’ judgments of their capabilities and are consolidated through repeated mastery experiences and the interpretation of feedback (Bandura, 1997). Within the teacher self-efficacy literature, the quality of classroom interaction and students’ responses are frequently treated as salient informational sources for efficacy appraisals (Tschannen-Moran and Hoy, 2001). In this study, teachers’ accounts of “smoother lessons,” “the transfer of rule awareness,” and the development of teacher–student “tacit coordination” can be interpreted as perceived gains in classroom controllability and accumulations of mastery experiences. Such experiences plausibly support more positive evaluations of teaching capability.

In addition, teachers highlighted that Orff-oriented lessons often involve performance-based and process-oriented activities, rendering students’ learning and participation more “visible.” This visibility appears to facilitate timely observation, documentation, and targeted instructional adjustment. The finding resonates with research on teacher professional learning and reflective practice, which emphasizes that teachers develop more stable instructional judgments through reflection on classroom interaction, immediate feedback, and iterative pedagogical experimentation (Marschall, 2022; Wyatt, 2014), and that sustained reflection may contribute to more stable beliefs and professional confidence (Bayer et al., 2009; Flores and Day, 2006). Teachers’ reports of exchanging experience with colleagues and adjusting activities across classes and student profiles also echo research on collaborative professional learning and reflective practice (Gnawali, 2008; Locke, 2016; Xiaoyu, 2024). Taken together, the present study extends a general claim that Orff Schulwerk may support self-efficacy by offering a more fine-grained, process-oriented account: when feedback is observable and the consequences of instructional attempts can be interpreted, teachers may be more likely to accumulate mastery experiences that strengthen efficacy beliefs.

### 4.3 Work engagement in relation to Orff-informed practice (RQ3)

Teachers’ accounts further described work engagement in relation to Orff-informed practice, particularly through positive classroom affect, renewed teaching enthusiasm, and perceived instructional accomplishment. Conceptually, work engagement is commonly defined as a positive, fulfilling work-related psychological state characterized by vigor, dedication, and absorption (Schaufeli et al., 2002, 2006). In this study, teachers’ descriptions of “immersion,” “enjoyment,” and “time passing quickly” are consistent with engagement research that links positive affect, absorption-like experiences, and dedication to one’s work. Prior work also suggests stable associations between positive work experiences and work engagement, often positioned as a psychological process contrasting with burnout (Bakker et al., 2007).

Regarding teaching enthusiasm, teachers framed Orff practice as a source of motivation for sustained learning and instructional exploration, including reading, writing teaching cases, and integrating Orff ideas with other approaches (e.g., Kodály-related strategies or Curwen hand signs). These accounts reflect behavioural expressions often aligned with dedication and absorption, and they speak to music education scholarship that highlights the reciprocal relationship between creative teaching and professional growth (Biasutti and Concina, 2018; Concina, 2023). In terms of accomplishment, teachers frequently attributed their sense of achievement to students’ immediate participation, positive feedback, and post-class interaction. This points to teacher–student interaction and co-creation as a potentially important situational basis for engagement experiences. Related studies similarly indicate that high-quality interaction and co-creative processes in music classrooms may support teachers’ occupational well-being and positive work experiences (Pellegrino, 2015; X. Wang, 2022).

Taken together, teachers’ narratives suggest that positive affect, renewed enthusiasm for teaching, and student-feedback-based accomplishment often co-occurred and jointly constituted a subjective foundation for higher engagement. These findings provide contextualized qualitative evidence of how teachers made sense of their engagement through concrete classroom activities and interactional processes.

The JD–R model posits that job demands (e.g., time pressure, emotional labour, and workload) can increase burnout risk through an exhaustion process, whereas job resources (e.g., autonomy, supportive relationships, and development opportunities) promote work engagement through a motivational process and buffer the adverse effects of high demands (Bakker and Demerouti, 2007; Demerouti et al., 2001; Hakanen et al., 2006). The model also highlights the critical role of personal resources—such as self-efficacy, optimism, and organization-based self-esteem—which are positively associated with work engagement and may amplify the benefits of job resources or mitigate the impact of high demands (Xanthopoulou et al., 2007). Interpreted through this lens, the classroom features of Orff Schulwerk and its supportive ecology (e.g., training opportunities, collegial teaching-research exchange, and positive student feedback) can be conceptualized as a constellation of potential job resources. These resources may enhance interaction quality and the visibility of feedback, strengthen perceived classroom controllability and instructional confidence, and thus create conditions that are conducive to engagement. In parallel, increased teaching self-efficacy can be understood as growth in personal resources that further sustains higher engagement. This pathway is consistent with the JD–R “resources–motivation” process and aligns with evidence linking personal resources (e.g., self-efficacy and resilience) to teachers’ work engagement (Sheridan et al., 2025).

The present data also suggest that resource effects are contingent on contextual conditions. When teachers have not yet developed proficient strategies for activity organization, or when external support is limited (e.g., insufficient training or weak school-based teaching-research support), some teachers may remain at an early stage marked by “infrequent use” or “lack of proficiency.” This implies that a pedagogy does not automatically translate into sustainable resource experiences. Such a pattern is consistent with the JD–R view that the availability of resources and the intensity of demands jointly shape whether motivational processes can be initiated and maintained (Bakker and Demerouti, 2007). Accordingly, the findings point to the need to attend to the broader school ecology that enables Orff Schulwerk to function as a lived resource for teachers.

### 4.4 Convergences and contextual specificities relative to prior research

Situating the present findings within prior Orff Schulwerk research surfaces both convergences with international literature and features specific to the Chinese setting.

Convergences with international research. Three patterns reported by our participants align closely with findings from Orff-related studies in other educational systems. First, teachers’ descriptions of an experiential, multimodal, and child-centred pedagogical orientation echo accounts from contexts as varied as Turkey, Aotearoa New Zealand, and the United States, where Orff-trained teachers similarly report increased emphasis on student-active, exploration-based classroom organization (Locke, 2016; Ozevin, 2021; Sogin and Wang, 2008). Second, our participants’ accounts linking Orff-informed practice to perceptions of greater classroom controllability and instructional confidence resonate with prior work suggesting that Orff training is associated with stronger movement-related and classroom-enactment self-efficacy (Fisher and Rose, 2011; Kikoler, 2022). Third, teachers’ linkage of student engagement to their own affective and motivational experiences is consistent with international literature documenting reciprocal teacher–student affect transmission in music classrooms (Frenzel et al., 2018; Pellegrino, 2015). These convergences should be interpreted as descriptive parallels rather than as evidence of cross-context generalizability, given the small purposive sample.

Contextual specificities of the Chinese setting. Three features of teachers’ accounts were less prominent or framed differently in international literature. First, jiaoyan—the institutionalized, regularly scheduled school-based teaching-research activities that are a routine feature of Chinese basic education—was repeatedly framed by our participants as a primary route of professional learning, alongside formal training. Although collaborative teacher learning is well documented internationally, the routinized character of jiaoyan appears to play a particularly salient role in mediating teachers’ uptake and ongoing refinement of Orff Schulwerk in Chinese primary schools. Second, several teachers framed the experiential and movement-based features of Orff Schulwerk against the backdrop of large class sizes (commonly 40–55 students) and performance-oriented expectations from school administration and parents, structural conditions less prominent in much of the Western primary school context and consequential for whether teachers can sustain Orff-informed practice. Third, although prior Chinese-context studies have documented the localized adaptation of Orff Schulwerk through Chinese musical materials (Lisha and Banchongsilpa, 2025; L. Wang and Odena, 2025), our participants additionally highlighted the deliberate integration of Orff elements with other pedagogies (notably Kodály-related strategies and Curwen hand signs) as a professional strategy responsive to local curricular demands.

Challenges as boundary conditions on resource conversion. The challenge accounts consolidated in Section 3.4 also bear on the mechanisms proposed above. Read in JD–R terms, teachers’ narratives indicate that Orff Schulwerk did not become an occupational resource automatically. Before the approach was experienced as supportive of manageability or engagement, teachers first had to work through an unstable early phase, and the calibration work that followed never fully ended; both functioned as demands in their own right. Structural conditions imposed further limits, particularly the partial fit with prescribed textbooks and the composition of large classes. What emerges is a conditional-resource interpretation: the same pedagogy that teachers found energizing once routines had stabilized was, in its early stages, a source of disorder and extra workload. The trajectory from “mess” to “tacit coordination” described by several teachers also implies a timing effect. Institutional supports such as protected jiaoyan time and collegial exchange may matter most precisely during this conversion window, before routines have stabilized, and longitudinal designs could examine this directly. Because the purposive sample contains no discontinuation accounts, the failure pathway, in which the conversion never completes, remains undocumented; mapping it is a clear task for future research.

Theoretical and practical implications. Theoretically, these findings suggest that the appropriation of a pedagogical approach as a lived occupational resource is shaped not only by individual training but also by the institutional conditions—jiaoyan structures, class-size constraints, and the scope of teachers’ professional discretion—that mediate teachers’ day-to-day enactment. The findings thus extend the JD–R model’s notion of “job resources” by foregrounding the institutional ecology through which a pedagogy is converted into a usable resource. Practically, the findings indicate that policies promoting Orff Schulwerk in Chinese primary schools may be more sustainable when paired with structural supports: protected time for jiaoyan-based reflection, manageable class sizes, and access to ongoing rather than one-off training. For teacher educators, the findings underscore the value of designing Orff-related professional learning that explicitly addresses adaptation to local curricular and structural conditions, rather than transposing Western implementation models without modification.

### 4.5 Limitations and future directions

Several limitations of this study should be considered when interpreting the findings, and several have already been incorporated into the body of the Discussion (Sections 4.2–4.4) because they bear directly on interpretation.

First, the sample comprised 18 primary school music teachers from Shandong Province, China, recruited through purposive and snowball sampling on the basis of prior Orff-related teaching experience. While thematic saturation was reached during analysis, this design implies self-selection bias in at least two respects. Participants were teachers who had remained engaged with Orff Schulwerk long enough to be willing to reflect on it in depth, plausibly favouring more receptive and successful practitioners; teachers who had attempted Orff-informed teaching and discontinued it, or who held more critical views, are likely under-represented. As a result, the salience of positive experiences in our findings should be interpreted with caution. Negative experiences, sustained failures, and resistance from teachers or institutions are likely under-described relative to their actual prevalence in the wider population of Chinese primary music teachers.

Second, the study relied primarily on teachers’ self-reported accounts collected through semi-structured interviews. While interviews offer in-depth insight into how teachers interpret and construct meaning from their experiences, self-report data are susceptible to social desirability bias, recall bias, and strategic self-presentation, all of which may amplify the salience of positive experiences and attenuate reports of difficulty, ambivalence, or failure (Krumpal, 2013). The interviewer’s own positionality as a music-education researcher with prior Orff exposure may also have invited responses oriented toward the researcher’s perceived interests, even though open prompts and reflective memos were used to mitigate this risk.

Third, interviews were conducted via online audio calls, which facilitated participation across geographically dispersed schools but reduced access to nonverbal cues, classroom contextual details, and the interactional micro-processes that would be visible in classroom observation. The mechanism-oriented interpretations offered in the Discussion are therefore grounded mainly in narrative evidence, and the proposed pathways are best understood as hypotheses to be tested with direct observational and triangulated data in future research (Archibald et al., 2019).

Fourth, thematic analysis involves interpretive work in coding, theme development, and naming. Although two researchers independently coded all transcripts and resolved disagreements through structured consensus discussion, and although NVivo memos preserved a complete audit trail, the resulting themes inevitably reflect the analytic team’s framing and priorities. Future studies could strengthen the evidentiary chain by incorporating triangulation across multiple data sources and analyst teams (Nowell et al., 2017).

Fifth, potential institutional and structural conditions that may shape broader implementation of Orff Schulwerk—such as district-level assessment regimes, administrative workload, the allocation of music-specialist staffing across schools, and variation in school-based professional learning support—were not directly observed and could only be inferred from teachers’ accounts. Comparative research at the school- and system-level is needed to characterize these conditions more directly.

Future research could extend this work in several directions. First, multi-site research across broader regions and different school types, ideally with longitudinal designs, would help examine the stability and contextual contingency of the associations described here. Second, mixed-methods designs that integrate qualitative interviews with validated quantitative measures of teaching self-efficacy and work engagement would allow for both descriptive richness and inferential testing of the pathways generated by the present study. Third, incorporating additional data sources—classroom observations, lesson plans, student work artefacts, and school-based jiaoyan materials—would enable methodological triangulation and stronger inferential warrant. Finally, comparative work on different models of pedagogical integration (Orff alone, Orff combined with Kodály, Orff combined with other approaches) and their longer-term implications for teachers’ professional growth, teaching self-efficacy, and work engagement would help map the conditions under which Orff-informed practice is most likely to function as a sustainable occupational resource.

## 5 Conclusion

Drawing on semi-structured interviews with 18 primary school music teachers in Shandong Province, China, and informed by self-efficacy theory and the Job Demands–Resources model as sensitizing frameworks, this study generates contextualized qualitative evidence on how Orff Schulwerk is enacted in Chinese primary music classrooms and how it is described in relation to teachers’ teaching self-efficacy and work engagement.

Teachers commonly encountered and developed their understandings of Orff Schulwerk through formal training, school-based jiaoyan teaching-research activities, and peer learning, and translated its principles into experiential, stepwise lesson routines. In teachers’ accounts, Orff-informed practice co-occurred with perceived growth in teaching self-efficacy—manifested as enhanced classroom manageability, greater instructional confidence, more visible learning assessment, and sustained reflection and adjustment based on student feedback—and with higher work engagement—manifested as more positive classroom affect, strengthened teaching enthusiasm, and a heightened sense of instructional accomplishment.

Taken together, teachers’ narratives suggest that Orff Schulwerk and its supportive school ecology may be experienced as salient occupational resources, and that perceived growth in personal resources such as self-efficacy may accompany the use of this pedagogy. These pathways are best understood as plausible mechanisms generated from teachers’ accounts, to be tested in future mixed-methods and longitudinal research. The findings carry implications for teacher professional development in Chinese primary music education: structural supports such as protected time for jiaoyan, manageable class sizes, and access to ongoing training appear to be conditions under which Orff Schulwerk is most likely to function as a sustainable occupational resource.

## Data Availability

The raw data supporting the conclusions of this article will be made available by the authors, without undue reservation.
