# Supplementary material for: Orff Schulwerk in Chinese primary music classrooms: a qualitative study of teachers’ teaching self-efficacy and work engagement
Source: Front Psychol. 2026 Aug 25;17:1838857. doi: 10.3389/fpsyg.2026.1838857 (PMC13551581; doi:10.3389/fpsyg.2026.1838857)
Supplement: Supplementary file 1 [file Supplementary_file_1.doc]

**Interview Guide**

1. Could you briefly introduce your educational background, age, and the number of years you have been teaching music?
2. Have you taught exclusively at the primary-school level, or have you also had teaching experience at other educational stages?
3. Could you briefly describe the size of the school where you currently work? Approximately how many music teachers are employed there?
4. Is your school a public school or a private school?
5. In addition to teaching music, do you hold any other responsibilities at the school, such as serving as a homeroom teacher, curriculum coordinator, or in an administrative role?
6. How many music lessons do you teach per week?
7. How did you first come into contact with, or learn about, the Orff Schulwerk approach?
8. How widely is the Orff Schulwerk approach used in your school? Do most music teachers use this approach, or do they combine it with other music teaching methods?
9. Drawing on your own teaching experience, how do you understand the Orff Schulwerk approach? In your view, what are its core principles?
10. How do you usually apply the Orff Schulwerk approach in your music teaching?
11. Could you describe, using a specific music lesson as an example, how you typically design and organize the different stages of the lesson?
12. When using the Orff Schulwerk approach, do you adapt the teaching content, activity design, or implementation methods according to your own teaching experience and the needs of particular classes? If so, in what ways do you usually make such adaptations?
13. Could you describe how students typically respond when participating in the Orff-based activities you design? Compared with more traditional music lessons, have you observed any changes in their interest, classroom participation, interaction, or learning engagement? Could you provide a specific example?
14. What attitudes do school leaders and parents generally hold toward teachers’ use of the Orff Schulwerk approach in music classes? Do they provide support in areas such as curriculum implementation, teaching resources, or extracurricular activities? Could you describe this based on your own experience?
15. Since implementing the Orff Schulwerk approach in your classes, have you found classroom management easier or more manageable? For example, have you noticed any changes in organizing student activities, maintaining classroom order, or sustaining students’ attention?
16. Compared with the period before you used the Orff Schulwerk approach, do you think the overall classroom atmosphere has changed? For example, have there been noticeable differences in student interaction, classroom liveliness, willingness to participate, or collaboration?
17. When assessing students’ learning processes and outcomes, do you feel that the Orff Schulwerk approach makes it easier to observe and evaluate their learning performance? For example, are you better able to notice changes in students’ musical skills, classroom participation, collaborative ability, creative expression, or learning efficiency? Could you provide an example?
18. From your personal teaching experience, since using the Orff Schulwerk approach, do you feel that your ability to manage lesson pacing, organize teaching, and teach with confidence has improved? Compared with before, do you feel more in control of the classroom and more confident in your teaching? Could you share a specific example?
19. When you complete an Orff-based music lesson successfully and see students participating actively and having positive learning experiences, do you feel a strong sense of achievement or satisfaction? Does this type of classroom experience further influence your enthusiasm for and engagement in teaching?
20. Would you be willing to share your experience of using the Orff Schulwerk approach with colleagues in your teaching-research group or teaching team, and encourage more teachers to learn about, try, or promote this approach? In what ways might you participate?
21. During your use of the Orff Schulwerk approach, have you found your teaching to become more interesting and dynamic? Has this change increased your own interest in, or anticipation of, teaching? Could you describe your experience?
22. When you are under considerable work pressure or feel mentally and physically tired, can a well-conducted Orff-based music lesson, in which students participate actively, help to relieve your emotions, restore your energy, or rekindle your enthusiasm for teaching? Could you describe this based on your own experience?
23. How do you understand the relationship between teachers’ teaching self-efficacy and work engagement? In your own teaching experience, do you think confidence in your teaching ability affects the enthusiasm, energy, and persistence you devote to teaching? Conversely, do you think higher work engagement can further strengthen your teaching confidence?
24. When you see students making clear progress in an Orff-based lesson, or showing strong enthusiasm and active participation, how do you usually feel? Does this positive classroom feedback further strengthen your willingness to continue using, exploring, and improving the Orff Schulwerk approach? Could you share a specific example?
25. Have you ever experienced a situation in which you carefully prepared an Orff-based music lesson, but the actual classroom outcome did not meet your expectations? In such cases, how do you usually reflect after class, and what adjustments do you make to subsequent teaching content, activity design, or classroom organization?
26. When you are in a positive state and highly enthusiastic about teaching, do you find it easier to respond to teaching difficulties or unexpected classroom situations by quickly identifying solutions and flexibly adjusting your teaching strategies?
27. What difficulties or challenges have you encountered while using the Orff Schulwerk approach? How do you usually address them?
28. Compared with traditional music teaching methods, what do you see as the main advantages and limitations of the Orff Schulwerk approach? Could you discuss this in relation to your own teaching experience?
29. In your view, what kinds of support could schools and educational authorities provide to better facilitate the implementation and promotion of the Orff Schulwerk approach in schools?
30. What advice or reminders would you offer to early-career music teachers, or teachers who are not yet familiar with the Orff Schulwerk approach? Based on your years of teaching experience, are there any practical strategies or lessons that could help them begin to try and apply this approach more effectively?
